# Pomegranate exerts chemoprevention of experimentally induced mammary tumorigenesis by suppression of cell proliferation and induction of apoptosis

**DOI:** 10.1080/01635581.2016.1115094

**Published:** 2015-12-23

**Authors:** Anupam Bishayee, Animesh Mandal, Piyali Bhattacharyya, Deepak Bhatia

**Affiliations:** ^a^ Department of Pharmaceutical Sciences, College of Pharmacy, Larkin Health Sciences Institute, Miami, Florida, USA; ^b^ Department of Pharmaceutical Sciences, College of Pharmacy, Northeast Ohio Medical University, Rootstown, Ohio, USA; ^c^ School of Health Sciences, University of Turabo, Gurabo, Puerto Rico; ^d^ Department of Pharmacogenomics, Bernard J. Dunn School of Pharmacy, Shenandoah University, Ashburn, Virginia, USA

## Abstract

Breast cancer is the second leading cause of cancer-related death in women in the United States and discovery and development of safe chemopreventive drugs is urgently needed. The fruit pomegranate (*Punica granatum*) is gaining importance because of its various health benefits. This study was initiated to investigate chemopreventive potential of a pomegranate emulsion (PE) against 7,12-dimethylbenz(*a*)anthracene (DMBA) rat mammary carcinogenesis. The animals were orally administered with PE (0.2–5.0 g/kg), starting 2 wk before and 16 wk following DMBA treatment. PE exhibited a striking reduction of DMBA-induced mammary tumor incidence, total tumor burden, and reversed histopathological changes. PE dose-dependently suppressed cell proliferation and induced apoptosis in mammary tumors. Immunohistochemical studies showed that PE increased intratumor Bax, decreased Bcl2 and manifested a proapoptotic shift in Bax/Bcl2 ratio. In addition, our gene expression study showed PE-mediated upregulation of Bad, caspase-3, caspase-7, caspase-9, poly (ADP ribose) polymerase and cytochrome *c* in mammary tumors. Thus, PE exerts chemoprevention of mammary carcinogenesis by suppressing cell proliferation and inducing apoptosis mediated through upregulation of Bax and downregulation of Bcl2 in concert with caspase cascades. Pomegranate bioactive phytoconstituents could be developed as a chemopreventive drug to reduce the risk of breast cancer.

## Introduction

Breast cancer is the most frequently diagnosed cancer and the primary cause of cancer-related death in women worldwide (1). The highest breast cancer incidence rates have been reported in North America, Western and Northern Europe, as well as Australia and the incidence and mortality of breast cancer have been rising in low- to middle-income countries (1). In the United States, an average woman has a 1 in 8 lifetime risk of breast cancer (2). About 233,000 new cases of breast cancer and 40,000 deaths due to this disease have been estimated to occur in women in the United States in 2014 (3). The estimated cost of breast cancer management in the United States is about 16.5 billion dollars per year and this cost is more than any other cancer (4).

Risk factors of breast cancer include age, family history, and genetic abnormalities, such as mutations in tumor suppressor genes *BRCA1* and *BRCA2* (5). Other risk factors are related to hormones, including early age of menarche, late onset of menopause, nulliparity, late age of first parity, lactation, use of oral contraceptive and hormone replacement therapy (6,7). Moreover, various life style-related factors, such as alcohol consumption and obesity, and disease conditions (e.g., diabetes), also contribute to the occurrence of breast cancer (7–10).

In view of limited treatment options for advanced stage breast cancer, preventing the development of breast cancer represents the most effective and prudent measure to reduce the mortality and morbidity. According to a recent consensus, preventive therapy needs to be integrated into wider strategies of breast cancer risk reduction, including weight control and increase in physical activity (11). Tamoxifen and raloxifene have been shown to reduce the risk of developing primary invasive breast cancer in high-risk women and accordingly approved by the United States Food and Drug Administration. Nevertheless, the use of the aforementioned medications is limited due to concern about adverse effects, including endometrial cancer, cataract and thromboembolic events, and poor ability to identify women at high risk (12,13). In view of these limitations, discovery and development of newer breast cancer chemopreventive drugs with acceptable efficacy and toxicity is urgently needed (14).

An increased understanding of the correlation between healthy diet and reduced incidence of cancer, including breast cancer, has led researchers to investigate breast cancer preventive effects of dietary natural products (15–18). We and other investigators have provided convincing evidence that various dietary agents and bioactive phytoconstituents prevent the occurrence of breast tumors or suppress the growth of existing tumors through modulation of cell proliferation, cellular differentiation, apoptosis, angiogenesis and several signaling pathways (19–21). Nevertheless, breast cancer preventive effects of a large number of dietary agents and phytochemicals are either not investigated or not fully understood.

Pomegranate (*Punica granatum*, L.) fruit is widely consumed fresh as well as in processed forms, such as juice, jams, sauce, and wine. Pomegranate, a native of the Himalayas in northern India, has been cultivated and naturalized throughout the Middle East, Mediterranean region, arid regions of Southeast Asia, tropical Africa, and the drier areas of the United States, including California, Arizona, and Texas. Known as “a pharmacy unto itself” in the Ayurvedic and Unani systems of medicine, pomegranate has been used for centuries for the prevention and treatment of a large numbers of ailments, including inflammation, diabetes, diarrhea, dysentery, dental plaque, intestinal infection, and malaria (22). Pomegranate has been gaining popularity as a functional food due to reports on potential health benefits, such as prevention and/or treatment of cancer, cardiovascular disease, obesity, diabetes, inflammation, ulcer, arthritis, microbial infection, acquired immune deficiency syndrome, neurological disorders, and erectile dysfunction and male infertility (23–26). Pomegranate juice is known to possess superior antioxidant property to that of other common fruit juices and this effect has been linked to the presence of polyphenols (27). Several pharmacological effects of pomegranate are related to a large number of phytochemicals, including hydrolyzable tannins and related compounds (ellagitanin, punicalagin, pedunculagin, punicalin, gallagic acid, ellagic acid and gallic acid), flavonoids (anthocyanins and catechins), flavonols (quercetin and kaempferol), flavones (apigenin and luteolin), and conjugated fatty acids (punicic acid), present in discrete anatomical parts, such as peel (pericarp or husk), juice, and seeds (26–28). Pomegranate juice, extracts, and phytoconstituents have been extensively studied preclinically for their anticarcinogenic and cancer chemopreventive effects in colon, lung, skin, and prostate cancer (26,28,29). Interestingly, investigation on pomegranate effects on breast cancer is limited mostly to in vitro studies. Several pomegranate products and phytochemicals inhibited the growth of both estrogen receptor-positive (MCF-7 and BT-474) and -negative (MB-MDA-231) breast carcinoma cell lines (30–35), mammary organ culture (36), MMTV-Wnt-1 mouse mammary cancer stem cells (37), suppressed the motility and invasion of aggressive breast cancer cells, e.g., MDA-231 and SUM 149 (38), and stimulated adhesion and inhibited the migration and chemotaxis (reminiscent of metastatic behavior) of MCF-7 and MDA-231 cells (39). Pomegranate juice concentrate was found to reduce the volume and weight of xenografted BT-474 tumors in athymic nude mice (34). There is no published report on in vivo chemopreventive effect of pomegranate against breast cancer to the best of our knowledge and belief. Accordingly, the objectives of the current study were (a) to evaluate the chemopreventive potential of a pomegranate formulation (emulsion) containing most bioactive constituents present in the whole fruit, and (b) delineate the possible mechanism(s) of action in a well-established, in vivo preclinical mammary carcinoma model utilizing female Sprague-Dawley rats and 7,12-dimethyl benz(*a*)anthracene (DMBA).

## Materials and methods

### Materials

Pomegranate emulsion (PE), a proprietary combination of pomegranate aqueous phase extract and seed oil, was purchased from Rimonest Ltd. (Haifa, Israel). Because this product contained various phytochemicals present in several parts (e.g., peels, juice, leaves, flowers and seeds) of the whole fruit, it is used for this study to maximize the synergy of pomegranate phytoconstituents. The detailed description on the preparation of this product was reported previously (40). The chemical analyses of this formulation revealed the presence of caffeic acid, corilagin, ellagic acid, ferulic acid, gallic acid, 5-hydroxymethylfurfural, protocatechuic acid, punicalagins (A and B) and *trans-p*-coumaric acid in the aqueous phase and mixed octadecatrienoic acids, sterols, and steroids, especially 17-α-estradiol, and tocol and γ-tocopherol in the lipid phase (40). DMBA, a mammary gland carcinogen, was procured from Sigma-Aldrich (St. Louis, MO). Paraformaldehyde was obtained from Ted Pella (Redding, CA). Primary antibodies, namely proliferating cell nuclear antigen (PCNA, sc-56), Bax (sc-70407), Bcl2 (sc-7382), and β-actin (sc-47778), were purchased from Santa Cruz Biotechnology (Santa Cruz, CA). TdT-FragEL™ DNA fragmentation detection kit was obtained from EMD Biosciences, Inc. (San Diego, CA). Quick RNA mini Prep kit and Verso cDNA synthesis kit were procured from Zymo Research (Irvine, CA) and Thermo Fisher Scientific (Waltham, MA), respectively.

### Animals and diet

The animal study was conducted at Northeast Ohio Medical University (NEOMED, Rootstown, OH) following an animal protocol approved by the NEOMED Institutional Animal Care and Use Committee in line with the *Guide for the Care and Use of Laboratory Animals* (NIH publication No. 85–23, revised in 1996). Pathogen-free virgin female Sprague-Dawley rats (approximately 36 days of age) were purchased from Harlan Laboratories (Indianapolis, IN) and housed in an animal facility accredited by the American Association for the Accreditation of Laboratory Animal Care. The rats were acclimatized to standard housing conditions, including ambient temperature of 22 ±2°C, relative humidity at 30–50%, and a 12-h light-dark cycle, in plastic cages (maximum 4 animals/cage) with special bedding (Cell-Sorb® Plus purchased from Fangman, Cincinnati, OH) for 1 wk before initiation of the experiment. The animals had free access to a well-defined, Constant Nutrition® formula basal rodent diet (Formulab 5008 from LabDiet, St. Louis, MO) and drinking water.

### Animal treatment protocol

**Figure 1.**
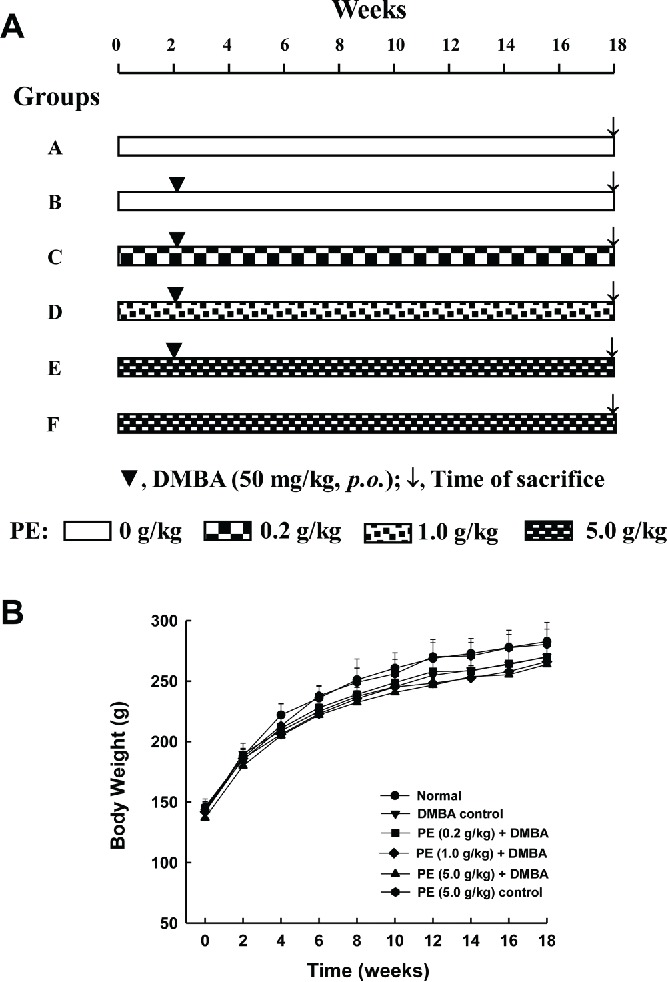
Experimental protocol and animal growth during the entire term of the study. A: Schematic representation of the experimental design utilized to investigate the effect of pomegranate emulsion (PE) on 7,12-dimethylbenz(*a*)anthracene (DMBA)-induced rat mammary carcinogenesis. B: Effect of dietary PE on body weight gain during DMBA-initiated mammary tumorigenesis in rats. Each data point indicates mean ± SEM (*n* = 12 for Group A, 11 for Group B, 8 each for Groups C and D, 7 for Group E, and 5 for Group F). No significant difference in body weights was observed among various rat groups at any time-point of the study.

The potential chemopreventive role of PE was investigated using a well-established and our previously published DMBA-induced rat mammary tumorigenesis model (41). The animal treatment protocol is depicted in Fig. 1A. Following 1-wk acclimatization period, the rats were divided into 6 groups. Two animal groups (Groups A and B) were maintained on the basal diet. The remaining 4 groups (Groups C–F) were fed with PE through oral gavage (p.o.) 3 times per wk (Monday, Wednesday, and Friday) in addition to being exposed to the basal diet. PE was administered by gently securing an animal by holding the skin at the back of its head and delivering the emulsion slowly via an animal feeding needle (Popper & Sons, Inc., New Hyde Park, NY). Three doses of PE were used, such as 0.2 g/kg (Group C) or 1.0 g /kg (Group D) and 5.0 g/kg (Groups E and F). These doses were selected based on our previous study (40). Following 2 wk of this feeding regimen and at approximately 57 days of age, mammary carcinogenesis was initiated in all animals belonging to Groups B, C, D, and E by a single administration of DMBA at 50 mg/kg body weight (dissolved in corn oil) by oral gavage according to our previous publication (41). The specific time for DMBA exposure is based on carcinogenic bioassay that indicates that rats at this age possess high frequency of terminal end buds that are more sensitive to DMBA in initiating mammary tumors (42). Feeding of rats with PE in Groups C, D, E and F were continued for another 16 weeks following the DMBA administration (i.e., a total period of 18 wk). Food and water intake as well as behavioral patterns were monitored daily and body weights of animals were recorded every other week. Palpation of mammary tumors (twice a week) began 4 wk following DMBA treatment. The experiment was terminated and all animals were sacrificed at 16 wk post-DMBA administration (i.e., 18 wk following the initiation of the experiment).

### Morphology and histopathology

Following an overnight fast, animals from each group were anesthetized by intramuscular injection of 60 mg/kg ketamine and 10 mg/kg xylazine. The skin was dissected out to expose mammary gland tumors. The tumors (approximated spheres) were separated from mammary gland parenchyma, carefully excised, rinsed with phosphate-buffered saline (pH 7.4) to flush out any residual blood, blotted dry on a paper towel, weighed, and photographed. Each mammary tumor was measured in 2 perpendicular directions to the nearest mm with a vernier caliper to obtain an average diameter. Photographs of tumors were captured by a digital camera (PowerShot ELPH520HS, Canon, Tokyo, Japan). The tumor incidence was calculated by dividing the number of rats with tumors by the total number of rats for each group and expressed as a percentage. The total tumor burden for each group represents the sum of individual tumor weights from all animals belonging to a group. The tumor burden values of PE treated groups were compared against that of control to calculate percentage inhibition.

The representative tumor tissue was harvested and cut into 2 halves. One half was immediately flash frozen in liquid nitrogen, subsequently transferred to −70 °C freezer and used for molecular work. The other half was fixed in 4% paraformaldehyde and utilized for histopathological and immunohistochemical analysis. The mammary tumors were classified according the established criteria (43). Transverse serial tumor sections (approximately 15-μm thick) were prepared using a microtome. Subsequently, these tissue sections were used for histopathological assessment by hematoxylin and eosin (H&E) staining.

### Immunohistochemical assessment

Serial sections of tumor tissue were utilized for immunohistochemical analysis. Cell proliferation was investigated by immunohistochemical detection of PCNA as a proliferation marker following our published method (44). Detection of apoptotic cells in tumor sections was performed by TdT-FragEL™ DNA fragmentation detection assay as described previously (44). The immunohistochemical analysis of Bax and Bcl2 was performed by methods we described elsewhere (44). The immunohistochemical slides were observed under a light microscope (BX43, Olympus, Center Valley, PA) and 1000 tumor cells/animal were analyzed. The PCNA labeling index (LI) was determined by counting the number of PCNA-positive cells × 100/total number of tumor cells. The apoptotic index (AI) was expressed as the number of positively stained tumor cells per 100 cells counted. All other immunohistochemical results were expressed as percentage of immunopositive cells.

### Reverse transcription-polymerase chain reaction

The mRNA expression levels of the apoptosis-related genes were analyzed by reverse transcription-polymerase chain reaction (RT-PCR) using gene-specific primers (45). Total RNA from 20 mg of tumor sample was extracted using Quick RNA mini Prep kit following the instructions provided by the vendor. The expression levels of proapoptotic and anti-apoptotic genes were monitored by RT-PCR using the cDNA verso kit with a temperature scale of 42°C for 30 min for reverse transcription, and 32 cycles of 94°C for 30 s, 56°C for 30 s, and 72°C for 30 s. The RT-PCR was carried out using the primers: BAD-F – 5′-GAGCTGACGTACAGCGTTGA-3′, BAD-R – 5′-GGGTAGGGTGTGTGGAAAAC-3′; BAX-F – 5′-AGGGGCCTTTTTGTTACAGG-3′, BAX-R – 5′-ACGTCAGCAATCATCCTCTG-3′; BCL2-F – 5′- CTTTGCAGAGATGTCCAGTCAG-3′, BCL2-R – 5′- AACTTTGTTTCATGGTCCATCC-3′; CASP3-F – 5′-AGGGTGCTACGATCCACCAGCA-3′, CASP3-R – 5′-CCATGGCTCTGCTCCGGCTC-3′; CASP7-F – 5′-GCCATGCCCAGGACAAGCCA-3′, CASP7-R – 5′-GCACGCCGGAGGACATGGTT-3′; CASP9-F – 5′- TGGGTCTCGGCGGGATCAGG −3′, CASP9-R – 5′- TGGCTGCTTGCCCACTGCTT −3′; poly (ADP ribose) polymerase (PARP)-F – 5′-CGACACGTTAGCGGAGCGGAC-3′, PARP-R – 5′-GCGCCCGCTCTTAGCGTACT-3′; CYTC-F - 5′- AGACTCACCCGTGCTTCAGT −3′, CYTC-R - 5′- ACTCCCAATCAGGCATGAAC-3′; and GAPDH-F – 5′-AGACAGCCGCATCTTCTTGT-3′, GAPDH-R – 5′-TACTCAGCACCAGCATCACC-3′. All primer sequences were designed using the Primer3 online program and synthesized by Eurofins MWG Operon (Huntsville, AL). The PCR products were analyzed on 1% agarose gel and visualized by ethidium bromide staining.

### Statistical analysis

Data are presented as mean ± SEM unless reported otherwise. The incidence of mammary tumor development in various groups was analyzed by Fisher's exact probability test. For other endpoint markers, significant differences among various groups were determined by one-way analysis of variance followed by Holm-Sidak test. A probability (*P*) level less than 0.05 was considered to be statistically significant. The commercial software SigmaPlot 11.0 (Systat Software, Inc., San Jose, CA) was used for all statistical analysis.

## Results

### General observations

No differences in water and food intakes were noticed among various experimental groups during the entire period of the study (18 wk). Likewise, no behavioral changes were observed among various animal groups. The growth pattern of animals remained unaffected by any treatment during the entire study since no significant difference was observed in the body weight between normal and any treated group at any time-point (Fig. 1B).

### PE inhibits DMBA-induced rat mammary tumorigenesis

While there were no visible tumors in the mammary glands of normal (Group A) as well as PE (5.0 g/kg) control (Group F) rats, macroscopic mammary tumors of various sizes and shapes were detected in the mammary glands of all DMBA-exposed groups. Table 1 summarizes data on mammary tumor incidence, total tumor burden and average tumor weight of DMBA-initiated groups with or without PE treatment. PE at a dose of 0.2 g/kg (Group C) or 1.0 g/kg (Group D) exhibited 20% reduction in tumor incidence compared to the DMBA control (group B), but the results did not reach the level of statistical significance. On the other hand, a significantly (*P* < 0.05) reduced tumor incidence (54%) was observed in the rat group that received PE at a dose of 5.0 g/kg (Group E) as compared to DMBA control (Group B). Oral PE treatment also reduced the total cumulative tumor burden (76–93%) in various DMBA-initiated groups in a dose-responsive fashion. The average tumor weight was found to be 86–90% smaller in all PE-treated groups compared to DMBA control (Group B). Interestingly, all these results were found to be statistically significant (*P* < 0.001).

**Table 1.** Effect of oral PE administration on DMBA-induced mammary tumorigenesis in Sprague-Dawley rats.

Treatment groups	No. of rats with tumors/total rats	Tumor incidence (%)	Total tumor burden (g)	Inhibition (%)	Average tumor weight (g)	Inhibition (%)
Group B: DMBA	9/11	82	89.0	—	14.8±4.8	—
Group C: PE (0.2 g/kg) + DMBA	5/8	62	21.7	76	2.0±0.3[Fn t1fn0002]	86
Group D: PE (1 g/kg) + DMBA	5/8	62	16.8	81	1.4±0.3[Fn t1fn0002]	90
Group E: PE (5 g/kg) + DMBA	2/7	28[Fn t1fn0001]	5.9	93	1.5±0.9[Fn t1fn0002]	90

Rats from normal (Group A, *n* = 12) and PE (5 g/kg) control group (Group F, *n* = 5) did not show any visible mammary tumor. PE = pomegranate emulsion; DMBA = 7,12-dimethylbenz(*a*)anthracene.

* *P* < 0.05 compared with DMBA (Group B) by Fisher's exact probability test.

** *P* < 0.001 compared with DMBA (Group B) by analysis of variance followed by Holm-Sidak test.

**Figure 2.**
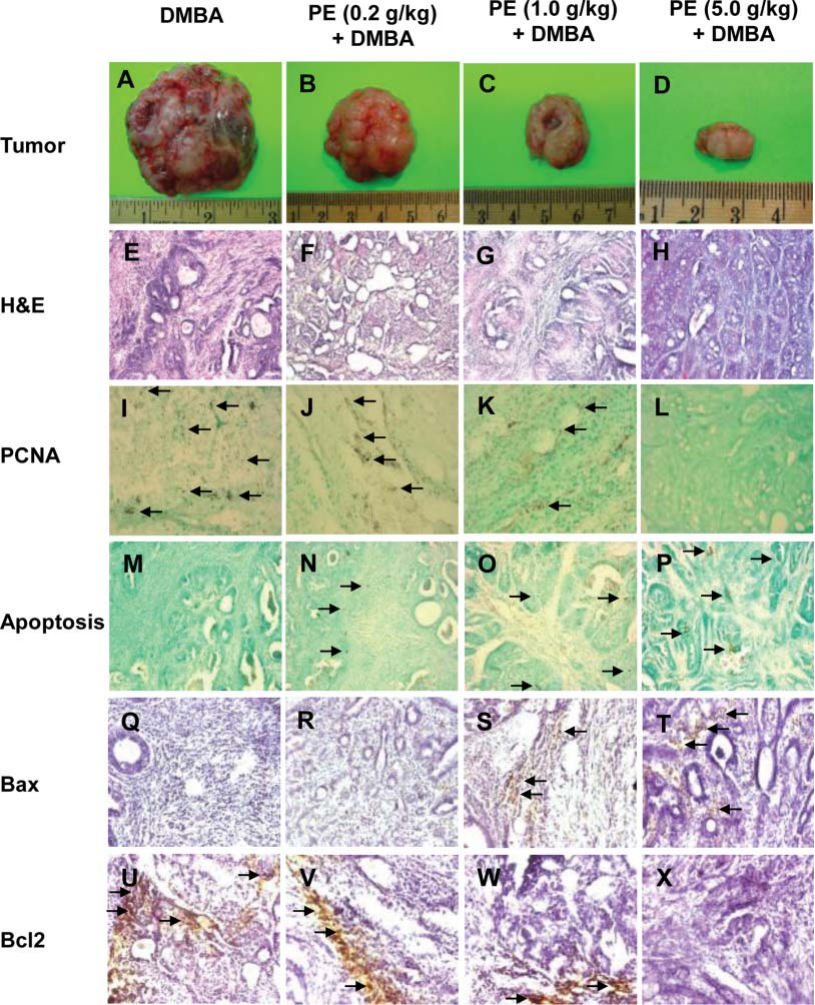
Chemoprevention of 7,12-dimethylbenz(*a*)anthracene (DMBA)-initiated rat mammary tumorigenesis by pomegranate. Effects of pomegranate emulsion (PE) on the size of mammary tumors (A–D), intratumor histopathological profiles (E–H), cell proliferation (I–L), apoptosis (M–P), Bax (Q–T), and Bcl2 (U–X) protein expression. The rats were treated with oral PE 2 wk prior to and 16 wk following DMBA administration. All animals were sacrificed 16 wk following DMBA exposure. The mammary tumors were subjected to morphological observation as well histopathological (H&E) and immunohistochemical analysis using anti-PCNA, anti-Bax, and anti-Bcl2 antibodies. Apoptosis was detected by DNA fragmentation assay. Magnification: 100× for tumor and H&E and 200× for PCNA, apoptosis, Bax, and Bcl2.

Most of the mammary tumors in rats treated with DMBA only were found to be large (Fig. 2A). Oral administration of rats with PE at low dose (0.2 g/kg) reduced the size of tumors in DMBA-treated animals (Fig. 2B). A substantial decrease in the size of tumor was observed in rats given PE at medium dose (1.0 g/kg) (Fig. 2C). Interestingly, the tumors from the high dose (5 g/kg) PE plus DMBA group exhibited remarkable reduction in size compared to those from any other DMBA-exposed animals (Fig. 2D).

### PE changes intratumor histopathological characteristics

Based on histopathological examination of H&E-stained mammary tumor sections, the majority of tumors in DMBA control rats showed extensive epithelial proliferation, resulting in a fused glandular pattern which indicates low-grade ductal carcinoma (adenocarcinoma). The cellular architecture exhibits substantial alteration and enlargement of the alveolus, presence of uniformly neoplastic ductal epithelial cells growing in cribriform pattern and nuclear pleomorphism, characterized by nuclear enlargement, prominent nucleoli and clumping of chromatids. Moreover, epithelial cells demonstrate gross variation in nuclear size and irregular chromatin (Fig. 2E). Although the low dose (0.2 g/kg) of PE did not alter intratumor histopathological features (Fig. 2F), a medium (1.0 g/kg) dose of PE exhibited a marked improvement of cellular architecture in mammary tumor tissue which indicates only mild hyperplasia (Fig. 2G). A further improvement of histopathological anomalies was observed with high dose (5.0 g/kg) of PE treatment (Fig. 2H). Tumor sections from this group showed almost normal ductal and alveolar structure with uniform epithelial cells without any sign of hyperplastic changes (Fig. 2H).

### PE inhibits tumor cell proliferation

**Figure 3.**
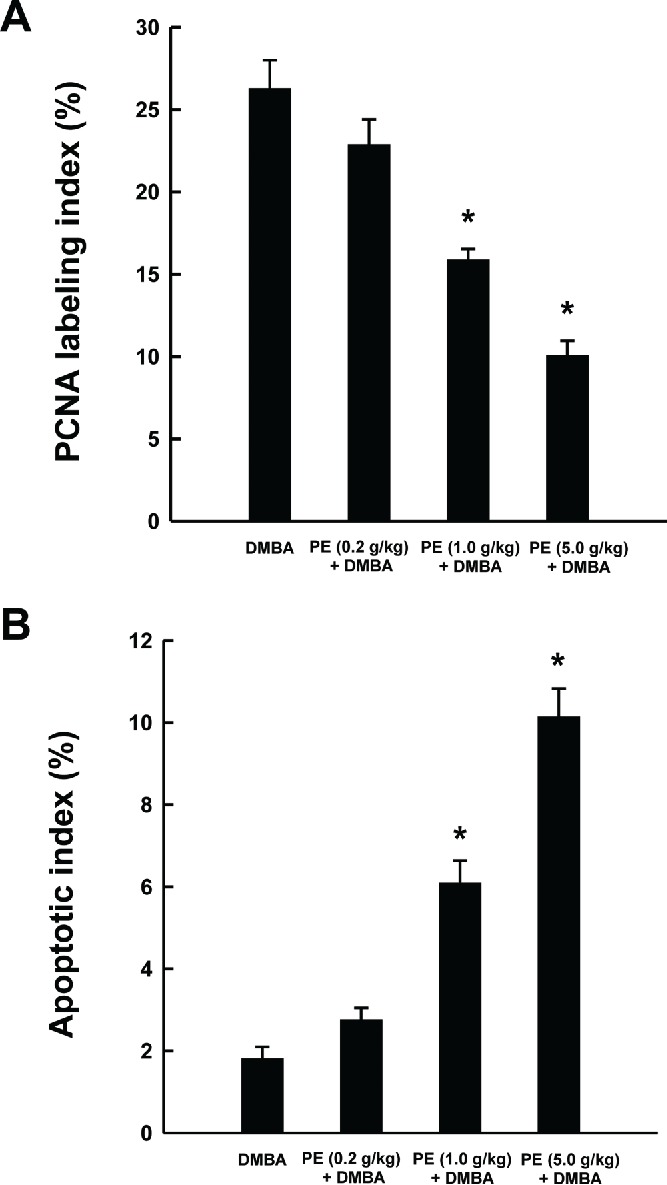
Quantitative analysis of mammary tumor cell proliferation and apoptosis during 7,12-dimethylbenz(*a*)anthracene (DMBA)-induced mammary carcinogenesis in rats in the presence or absence of pomegranate emulsion (PE). Effects of PE on intra-tumor PCNA LI as determined by immunohistochemistry (A) and apoptotic index (AI) as measured by DNA fragmentation assay (B). The labeling index (LI) or AI was expressed as the number of immunopositive cells 100×/total number of tumor cells analyzed. Results are expressed as mean ± SEM (*n* = 4). *P < 0.001 compared with DMBA control.

To determine whether PE impacts cellular proliferation in DMBA-induced mammary tumors, PCNA protein expression was assessed in serial tumor sections by immunohistochemical technique. Tumor sections from DMBA control rats showed an abundance of PCNA-positive cells, indicating extensive cell proliferation (Fig. 2I). Although a negligible reduction in proliferating tumor cells was observed in low dose PE group (Fig. 2J), a moderate and extensive suppression of cell proliferation was noticed in medium (Fig. 2K) and high dose PE group (Fig. 2L), respectively. As illustrated in Fig. 3A, the PCNA LI was found to be smaller in all PE-fed animals. Interestingly, a statistically significant (*P *< 0.001) reduction in PCNA LI was found in medium or high dose PE group exposed to DMBA compared to DMBA control group.

### PE exerts apoptosis in mammary tumors

We used TdT-FragEL™ DNA fragmentation detection assay to investigate the extent of programmed cell death (apoptosis) in mammary tumor samples. The chromagen-generated brown staining was indicative of apoptotic cells. The presence of apoptotic cells was sporadic in tumor samples harvested from DMBA control (Fig. 2M) and modest in low dose PE plus DMBA group (Fig. 2N). In contrast, we observed a large number of positive staining overlapping the condensed chromatin of apoptotic bodies in medium (Fig. 2O) or high dose PE plus DMBA group (Fig. 2P). Fig. 3B presents intratumor AI of all experimental groups. Although we did not observe any difference in AI between DMBA control and PE (low dose) plus DMBA group, there was a significant (*P* < 0.001) increase in AI in tumor sections obtained from 2 experimental groups that received PE at medium or high dose compared to DMBA alone.

### PE regulates apoptosis-related gene expressions

**Figure 4.**
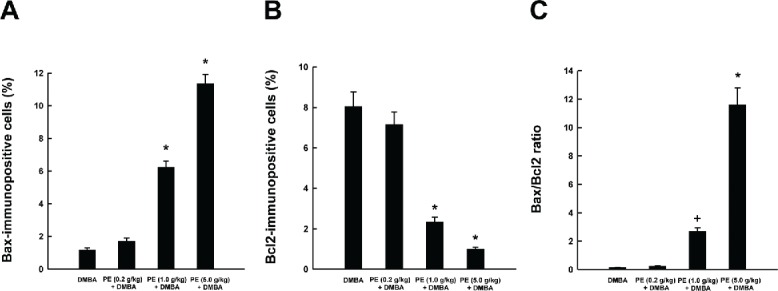
Effects of pomegranate emulsion (PE) on intratumor Bax and Bcl2 expression as determined by immunohistochemistry. Quantitative analysis of Bax-immunopositive cells (A), Bcl2-immunopositive cells (B), and Bax/Bcl2 ratio (C) in mammary tumors induced by 7,12-dimethylbenz(*a*)anthracene (DMBA) in rats. Each bar represents the mean ± SEM (*n* = 4). A,B: *P < 0.001; C: +*P* < 0.05 and *P < 0.001 as compared to DMBA control.

To better understand the possible mechanism(s) of apoptosis-inducing activity of PE, the expression of apoptosis-related proteins, such as Bax and Bcl2, was evaluated in mammary tumors by immunohistochemical technique. The occurrence of Bax-immunopositive cells was found to be extremely low in tumors sections harvested from DMBA control rats (Fig. 2Q) or low dose PE plus DMBA group (Fig. 2R). On the other hand, an obvious increase in the expression of Bax was noticeable in the cytoplasm of tumor sections obtained from medium (Fig. 2S) or high dose of PE (Fig. 2T). According to quantitative analysis, these 2 doses of PE significantly (*P* < 0.001) increased Bax-immunopositive cells in DMBA-initiated rats compared to DMBA control animals (Fig. 4A). The mammary tumor sections from DMBA control rats showed extensive expression of cytoplasmic Bcl2 protein (Fig. 2U) which was only slightly altered by the low dose of PE (Fig. 2V). Interestingly, other 2 doses of PE exhibited considerable reduction of Bcl2 immunopositive cells (Fig. 2W and 2X). The quantitative analysis of Bcl2-positive cells revealed a significant (*P* < 0.001) decrease in immunopositive cells in tumor sections from rats received medium or high dose of PE (Fig. 4B). Oral administration of PE before and after DMBA exposure elevated the Bax/Bcl2 ratio in a dose-responsive fashion (Fig. 4C). However, a statistically significant (*P* < 0.05 or 0.001) increase in of Bax/Bcl2 ratio was observed in the group that received the medium or high dose of PE compared to DMBA control, respectively.

**Figure 5.**
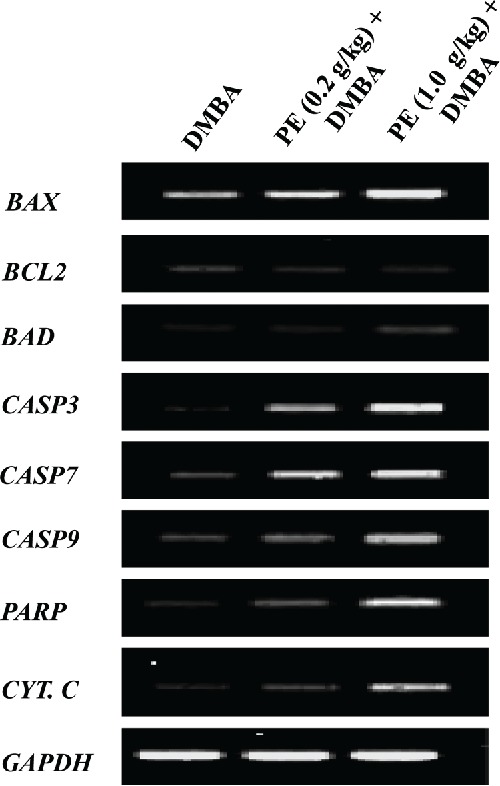
Effects of pomegranate emulsion (PE) treatment on transcriptional expressions of genes related to apoptosis in 7,12-dimethylbenz(*a*)anthracene (DMBA)-initiated rat mammary tumorigenesis. Rats were sacrificed 18 wk following the commencement of the study and mammary tumor samples were harvested from various experimental groups. Total RNA was extracted. The resultant complementary DNA following reverse transcription was subjected to PCR using specific sequences for various genes: *BAX, BCL2, BAD, CASPASE 3* (CASP3), *CASPASE 7* (CASP7), *CASPASE 9* (CASP 9), *poly (ADP-ribose) polymerase* (*PARP*), and *cytochrome c* (*CYT. C*). Representative reverse-transcriptase-PCR pictures are shown with *glyceraldehyde 3-phosphate dehydrogenase* (*GAPDH*) as the housekeeping gene.

Mammary tumor samples harvested from various experimental groups were used to perform RT-PCR to confirm our immunohistochemical data on apoptosis-related proteins. As depicted in Fig. 5, dietary treatment of PE at low and medium doses elevated the mRNA expression of *BAX* and reduced the mRNA expression of *BCL2* compared to DMBA control. In addition, a clear up-regulation of *BAD, CASP3, CASP7, CASP9, PARP*, and *CYT C* was noticed at transcriptional level due to TE administration. Most of these results showed a dose-responsive pattern. Our gene expression data clearly indicate a proapoptotic mechanism of PE-mediated prevention of rat mammary tumorigenesis.

## Discussion

The results of our study provide evidence for the first time that a pomegranate formulation (PE) exerts a striking chemopreventive activity against classical DMBA mammary tumorigenesis in female Sprague-Dawley rats. Although PE at low (0.2 g/kg) and medium dose (1.0 g/kg) registered inhibition of mammary tumor development in statistically insignificant manner, the tumor-inhibitory effect of PE at high dose (5.0 g/kg) was found to statistically significant. The potential chemoprotective response of PE was also reflected in the reduced total tumor burden and smaller average tumor weight in DMBA-initiated rats. Interestingly, all 3 doses of PE manifested reduction in average tumor weight in statistically significant manner. The chemopreventive effect of PE administration was also reflected in the results of our histopathological study that shows functional differentiation, decreased cell density and infiltration as well as non-invasiveness of tissue architecture due to PE treatment. The mammary tumor-inhibitory effect of PE against DMBA-initiated rats as observed in our present study is in agreement with several previously reported anti-breast cancer effects of pomegranate. For example, fermented pomegranate polyphenols, pomegranate juice polyphenols and pomegranate seed oil were found to substantially reduce (42–87%) the number of mammary lesions inflicted by DMBA in a murine mammary gland culture model (30,36). Moreover, an oral pomegranate juice concentrate significantly depressed the volume of BT474 xenografts in nude mice (34). Our present results on anti-breast carcinogenic effect of PE in vivo are in agreement with our previous findings that PE exhibits substantial chemopreventive action against chemically induced rat liver carcinogenesis through multiple cellular and molecular mechanisms (40,46,47).

Another interesting observation of our study was that PE treatment did not alter food intake, water intake, behavioral patterns, and growth rate of experimental animals. This finding may indicate that the observed chemopreventive effect of PE is devoid of any toxic manifestation. A prior subchronic toxicity study also showed that oral administration of a pomegranate extract at 600 mg/kg/d for 3 mo in rats did not exhibit any significant adverse effects based on body weight gains, feed consumption, organ weights, hematological, and gross histopathological evaluation (48).

To gain insight into the mechanism(s) by which PE abrogated mammary tumorigenesis in rats, we determined cellular proliferation in mammary tumors excised from DMBA-exposed rats with or without PE treatment. The extent of cell proliferation is routinely used in clinical situations for assessment of tumor prognosis and analysis of response of malignant cells to anticancer treatment (49). PCNA, a 36-kDa nuclear protein, functions as a cofactor for DNA polymerase δ and serves as an important proliferative marker for mammary carcinogenesis (50). In this study, we have used rat mammary tumor samples to analyze the expression of PCNA by immunohistochemical technique. An elevated expression of PCNA in mammary tumors of DMBA control animals indicates accelerated proliferation of tumor cells. Orally administered PE resulted in reduced expression of PCNA in conjunction with lower PCNA LI in tumor sections which strongly suggests antiproliferative mechanisms involved in the observed reduction of the incidence and growth of mammary tumors.

The progression of carcinogenesis selects against apoptosis to initiate, promote, and perpetuate the malignant phenotype and apoptosis-inducing ability is considered to be a major factor in evaluating the chemopreventive potential of a candidate agent. We have detected apoptosis in mammary tumor tissues by DNA fragmentation assay with immunohistochemistry. Our findings clearly demonstrate a substantial increase in DNA fragmentation with increasing doses of PE, indicating induction of apoptosis during mammary carcinogenesis. The higher frequency of apoptosis due to PE administration as observed in our study may curtail the progression of mammary carcinogenesis as reflected in the reduced incidence and growth of mammary tumors. Our observation on in vivo apoptosis-inducing effect of PE during mammary tumorigenesis is in accordance with other in vitro studies showing proapoptotic activities of pomegranate phytoconstituents in human breast cancer cells (30,32,35,38,51).

The activation of proapoptotic Bcl2 family members, such as Bax and Bad, stimulates apoptosis by causing pore formation in the mitochondrial membrane, resulting in the release of cytochrome *c* (cyt *c*). Subsequently, cyt *c* binds with apoptotic protease activating factor-1 which in turn binds to procaspase-9 to create a protein complex called apoptosome. The apoptosome cleaves the procaspase-9 to its active form caspase-9, which subsequently activates the effector caspase-3. Caspase-7, another downstream effector, is activated by both death receptor- (extrinsic) and mitochondrial-mediated (intrinsic) pathways. Activated caspase-3 and caspase-7 participate in a cleavage cascade of a number of cellular proteins, resulting in the characteristic biochemical and morphological hallmarks of apoptosis (52). Overexpression of Bcl2 promotes cell survival by suppressing apoptosis, whereas enhanced expression of Bax accelerated cell death. An increase in Bax/Bcl2 ratio is considered to be a reliable indicator of the overall propensity of a cell to undergo apoptosis. It is noteworthy that dysregulation of apoptosis due to imbalance in Bax/Bcl2 ratio has been implicated in the pathogenesis and progression of mammary gland carcinoma (53). In the current study, dietary treatment with PE increased Bax expression and decreased Bcl2 expression in mammary tumors induced by DMBA with a proapoptotic shift in Bax/Bcl2 ratio. Our gene expression study further supported immunohistochemical assessment of Bax and Bcl2. It also indicates transcriptional upregulation of Bad, cyt *c*., caspase-3, caspase-7, caspase-9, and PARP. All these results suggest that pomegranate bioactive phytoconstituents induce apoptosis in experimentally induced mammary tumors, at least, through mechanisms involving upregulation of proapoptotic genes, downregulation of antiapoptotic genes, and caspase cascade pathway.

The precise bioactive phytoconstituents of the pomegranate formulation responsible for the observed mammary tumor-suppressive and underlying mechanistic effects are not known at the present time and require additional thorough investigation. Various pomegranate phytochemicals present in the formulation used in this study showed synergistic interactions in inhibiting proliferation of and inducing apoptosis in human cancer cells (54,55). Hence, it is tempting to speculate that pomegranate phytochemicals may confer the observed antitumor, antiproliferative and proapoptotic activities through promotion of multifactorial effects and chemical synergy. One of the limitations of this study is that pomegranate emulsion was administered before, during, and after exposure to the carcinogen. Therefore, it is not possible to ascertain at what stage the pomegranate bioactive compounds are mostly active. However, it is possible that the observed effect may be due to either detoxification or reduced metabolic activation of DMBA.

In conclusion, the present study demonstrates for the first time that oral administration of PE 3 times a week for 18 wk exhibits a significant chemopreventive effect in DMBA classical rat model of chemically induced breast cancer. The dose-responsive chemopreventive effect of PE is evidenced from its ability to inhibit the development of mammary tumors, reduce tumor burden and alter tumor histopathological characteristics. Inhibition of abnormal cell proliferation and induction of apoptosis may explain, at least in part, the fundamental cellular mechanisms of mammary tumor-inhibitory efficacy of PE. Our data also suggest that downregulation of anti-apoptotic protein Bcl2 and upregulation of proapoptotic protein Bax in concert with caspase cascades may account for apoptosis-inducing activity of PE during DMBA-induced mammary carcinogenesis. All interesting results coupled with a safety profile may advance the development of pomegranate phytoconstituents as a complex chemopreventive drug to reduce the risk of breast cancer which is a complex disease.

## References

[cit0001] Jemal A, Bary F, Center MM, Ferley J, Ward E (2011). Global cancer statistics. *CA Cancer J Clin*.

[cit0002] American Cancer Society (2011). *Breast Cancer Facts and Figures 2011–2012*.

[cit0003] Siegel R, Ma J, Zou Z, Jemal A (2014). Cancer statistics, 2014. *CA Cancer J Clin*.

[cit0004] Yabroff KR, Lund J, Kepka D, Mariotto A (2011). Economic burden of cancer in the United States: estimates, projections, and future research. *Cancer Epidemiol Biomarkers Prev*.

[cit0005] Campeau PM, Foulkes WD, Tischkowitz MD (2008). Hereditary breast cancer: new genetic developments, new therapeutic avenues. *Hum Genet*.

[cit0006] Alberg AJ, Helzlsouer KJ (1997). Epidemiology, prevention, and early detection of breast cancer. *Curr Opin Oncol*.

[cit0007] Hanf V, Hanf D (2014). Reproduction and breast cancer risk. *Breast Care*.

[cit0008] Singletary KW, Gapstur SM (2001). Alcohol and breast cancer: review of epidemiologic and experimental evidence and potential mechanisms. *JAMA*.

[cit0009] Crujeiras AB, Díaz-Lagares A, Carreira MC, Amil M, Casanueva FF (2013). Oxidative stress associated to dysfunctional adipose tissue: a potential link between obesity, type 2 diabetes mellitus and breast cancer. *Free Radic Res*.

[cit0010] Pierobon M, Frankenfeld CL (2013). Obesity as a risk factor for triple-negative breast cancers: a systematic review and meta-analysis. *Breast Cancer Res Treat*.

[cit0011] Cuzick J, DeCensi A, Arun B, Brown PH, Castiglione M (2011). Preventive therapy for breast cancer: a consensus statement. *Lancet Oncol*.

[cit0012] Nelson HD, Smith ME, Griffin JC, Fu R (2013). Use of medications to reduce risk for primary breast cancer: a systematic review for the U.S. preventive services task force. *Ann Intern Med*.

[cit0013] Lazzeroni M, DeCensi A (2013). Breast cancer prevention by antihormones and other drugs: where do we stand?. *Hematol Oncol Clin North Am*.

[cit0014] Steward WP, Brown K (2013). Cancer chemoprevention: a rapidly evolving field. *Br J Cancer*.

[cit0015] Ogunleye AA, Xue F, Michels KB (2010). Green tea consumption and breast cancer risk or recurrence: a meta-analysis. *Breast Cancer Res Treat*.

[cit0016] Song JK, Bae JM (2013). Citrus fruit intake and breast cancer risk: a quantitative systematic review. *J Breast Cancer*.

[cit0017] Chajès V, Romieu I (2014). Nutrition and breast cancer. *Maturitas*.

[cit0018] Kado K, Forsyth A, Patel PR, Schwartz JA (2012). Dietary supplements and natural products in breast cancer trials. *Front Biosci*.

[cit0019] Bishayee A, Ahmed S, Brankov N, Perloff M (2011). Triterpenoids as potential agents for the chemoprevention and therapy of breast cancer. *Front. Biosci*.

[cit0020] Reuben SC, Gopalan A, Petit DM, Bishayee A (2012). Modulation of angiogenesis by dietary phytoconstituents in the prevention and intervention of breast cancer. *Mol Nutr Food Res*.

[cit0021] Vadodkar AS, Suman S, Lakshmanaswamy R, Damodaran C (2012). Chemoprevention of breast cancer by dietary compounds. *Anticancer Agents Med Chem*.

[cit0022] Ismail T, Sestili P, Akhtar S (2012). Pomegranate peel and fruit extracts: a review of potential anti-inflammatory and anti-infective effects. *J Ethnopharmacol*.

[cit0023] Jurenka JS (2008). Therapeutic applications of pomegranate (*Punica granatum* L.): a review. *Altern Med Rev*.

[cit0024] Faria A, Calhau C (2011). The bioactivity of pomegranate: impact on health and disease. *Crit Rev Food Sci Nutr*.

[cit0025] Johanningsmeier SD, Harris GK (2011). Pomegranate as a functional food and nutraceutical source. *Annu Rev Food Sci Technol*.

[cit0026] Viladomiu M, Hontecillas R, Lu P, Bassaganya-Riera J (2013). Preventive and prophylactic mechanisms of action of pomegranate bioactive constituents. *Evid Based Complement Alternat Med*.

[cit0027] Heber D, Benzie IFF, Wachtel-Galor S (2011). Pomegranate ellagitannins. *Herbal Medicine: Biomolecular and Clinical Aspects*.

[cit0028] Lansky EP, Newman RA (2007). *Punica granatum* (pomegranate) and its potential for prevention and treatment of inflammation and cancer. *J Ethnopharmacol*.

[cit0029] Adhami VM, Khan N, Mukhtar H (2009). Cancer chemoprevention by pomegranate: laboratory and clinical evidence. *Nutr Cancer*.

[cit0030] Kim ND, Mehta R, Yu W, Neeman I, Livney T (2002). Chemopreventive and adjuvant therapeutic potential of pomegranate (*Punica granatum*) for human breast cancer. *Breast Cancer Res Treat*.

[cit0031] Adams LS, Zhang Y, Seeram NP, Heber D, Chen S (2010). Pomegranate ellagitannin-derived compounds exhibit antiproliferative and antiaromatase activity in breast cancer cells in vitro. *Cancer Prev Res*.

[cit0032] Dikmen M, Ozturk N, Ozturk Y (2011). The antioxidant potency of *Punica granatum* L. fruit peel reduces cell proliferation and induces apoptosis on breast cancer. *J Med Food*.

[cit0033] Joseph MM, Aravind SR, Varghese S, Mini S, Sreelekha TT (2012). Evaluation of antioxidant, antitumor and immunomodulatory properties of polysaccharide isolated from fruit rind of *Punica granatum*. *Mol Med Rep*.

[cit0034] Banerjee N, Talcott S, Safe S, Mertens-Talcott SU (2012). Cytotoxicity of pomegranate polyphenolics in breast cancer cells in vitro and vivo: potential role of miRNA-27a and miRNA-155 in cell survival and inflammation. *Breast Cancer Res Treat*.

[cit0035] Shirode AB, Kovvuru P, Chittur SV, Henning SM, Heber D (2014). Antiproliferative effects of pomegranate extract in MCF-7 breast cancer cells are associated with reduced DNA repair gene expression and induction of double strand breaks. *Mol Carcinog*.

[cit0036] Mehta R, Lansk EP (2004). Breast cancer chemopreventive properties of pomegranate (*Punica granatum*) fruit extracts in a mouse mammary organ culture. *Eur J Cancer Prev*.

[cit0037] Dai Z, Nair V, Khan M, Ciolino HP (2010). Pomegranate extract inhibits the proliferation and viability of MMTV-Wnt-1 mouse mammary cancer stem cells in vitro. *Oncol Rep*.

[cit0038] Khan GN, Gorin MA, Rosenthal D, Pan Q, Bao LW (2009). Pomegranate fruit extract impairs invasion and motility in human breast cancer. *Integr Cancer Ther*.

[cit0039] Rocha A, Wang L, Penichet M, Martins-Green M (2012). Pomegranate juice and specific components inhibit cell and molecular processes critical for metastasis of breast cancer. *Breast Cancer Res Treat*.

[cit0040] Bishayee A, Bhatia D, Thoppil RJ, Darvesh AS, Nevo E (2011). Pomegranate-mediated chemoprevention of experimental hepatocarcinogenesis involves Nrf2-regulated antioxidant mechanisms. *Carcinogenesis*.

[cit0041] Bishayee A, Mandal A, Thoppil RJ, Darvesh AS, Bhatia D (2013). Chemopreventive effect of a novel oleanane triterpenoid in a chemically induced rodent model of breast cancer. *Int J Cancer*.

[cit0042] Russo J, Gusterson BA, Rogers AE, Russo IH, Wellings SR (1990). Comparative study of human and rat mammary tumorigenesis. *Lab Invest*.

[cit0043] Russo J, Russo IH (2000). Atlas and histologic classification of tumors of the rat mammary gland. *J Mammary Gland Biol Neoplasia*.

[cit0044] Bishayee A, Dhir N (2009). Resveratrol-mediated chemoprevention of diethylnitrosamine-initiated hepatocarcinogenesis: inhibition of cell proliferation and induction of apoptosis. *Chem Biol Interact*.

[cit0045] Floros KV, Thomadaki H, Florou D, Talieri M, Scorilas A (2006). Alterations in mRNA expression of apoptosis-related genes BCL2, BAX, FAS, caspase-3, and the novel member BCL2L12 after treatment of human leukemic cell line HL60 with the antineoplastic agent etoposide. *Ann N Y Acad Sci*.

[cit0046] Bishayee A, Thoppil RJ, Darvesh AS, Ohanyan V, Meszaros JG (2013). Pomegranate phytoconstituents blunt the inflammatory cascade in a chemically induced rodent model of hepatocellular carcinogenesis. *J Nutr Biochem*.

[cit0047] Bhatia D, Thoppil RJ, Mandal A, Samtani KA, Darvesh AS (2013). Pomegranate bioactive constituents suppress cell proliferation and induce apoptosis in an experimental model of hepatocellular carcinoma: role of Wnt/ β-catenin signaling pathway. *Evid Based Complement Alternat Med*.

[cit0048] Patel C, Dadhaniya P, Hingorani L, Soni MG (2008). Safety assessment of pomegranate fruit extract: acute and subchronic toxicity studies. *Food Chem Toxicol*.

[cit0049] Christov K, Grubbs C, Shikaitis A, Juliana MM, Lubet RA (2007). Short-term modulation of cell proliferation and apoptosis and preventive/therapeutic efficacy of various agents in a mammary cancer model. *Clin Cancer Res*.

[cit0050] Al-Dhaheri WS, Hassouna I, Al-Salam S, Karam SM (2008). Characterization of breast cancer progression in the rat. *Ann NY Acad Sci*.

[cit0051] Jeune MA, Kumi-Diaka J, Brown J (2005). Anticancer activities of pomegranate extracts and genistein in human breast cancer cells. *J Med Food*.

[cit0052] Chen N, Wang J (2002). Initiator caspases in apoptosis signaling pathways. *Apoptosis*.

[cit0053] Krajewski S, Krajewska M, Turner BC, Pratt C, Howard B (1999). Prognostic significance of apoptosis regulators in breast cancer. *Endocr Relat Cancer*.

[cit0054] Lansky EP, Jiang W, Mo H, Bravo L, Froom P (2005). Possible synergistic prostate cancer suppression by anatomically discrete pomegranate fractions. *Invest New Drugs*.

[cit0055] Seeram NP, Adams LS, Henning SM, Niu Y, Zhang Y (2005). In vitro antiproliferative, apoptotic and antioxidant activities of punicalagin, ellagic acid and a total pomegranate tannin extract are enhanced in combination with other polyphenols as found in pomegranate juice. *J Nutr Biochem*.

